# Kv1 potassium channels control action potential firing of putative GABAergic deep cerebellar nuclear neurons

**DOI:** 10.1038/s41598-020-63583-7

**Published:** 2020-04-24

**Authors:** Jessica Abigail Feria Pliego, Christine M. Pedroarena

**Affiliations:** 1 Graduate School of Cellular and Molecular Neurosciences, University of Tübingen, Tübingen, Germany; 2 Department for Cognitive Neurology, Hertie-Institute for Clinical Brain Research, 72076 Tübingen, Germany; 3 Systems Neurophysiology, Werner Reichardt Center for Integrative Neuroscience, University Tübingen, 72076 Tübingen, Germany

**Keywords:** Cellular neuroscience, Cerebellum

## Abstract

Low threshold voltage activated Kv1 potassium channels play key roles in regulating action potential (AP) threshold, neural excitability, and synaptic transmission. Kv1 channels are highly expressed in the cerebellum and mutations of human Kv1 genes are associated to episodic forms of ataxia (EAT-1). Besides the well-established role of Kv1 channels in controlling the cerebellar basket-Purkinje cells synapses, Kv1 channels are expressed by the deep cerebellar nuclear neurons (DCNs) where they regulate the activity of principal DCNs carrying the cerebellar output. DCNs include as well GABAergic neurons serving important functions, such as those forming the inhibitory nucleo-olivary pathway, the nucleo-cortical DCNs providing feed-back inhibition to the cerebellar cortex, and those targeting principal DCNs, but whether their function is regulated by Kv1 channels remains unclear. Here, using cerebellar slices from mature GAD67-GFP mice to identify putative GABAergic-DCNs (GAD + DCN) we show that specific Kv1 channel blockers (dendrotoxin-alpha/I/K, DTXs) hyperpolarized the threshold of somatic action potentials, increased the spontaneous firing rate and hampered evoked high frequency repetitive responses of GAD + DCNs. Moreover, DTXs induced somatic depolarization and tonic firing in previously silent, putative nucleo-cortical DCNs. These results reveal a novel role of Kv1 channels in regulating GABAergic-DCNs activity and thereby, cerebellar function at multiple levels.

## Introduction

The Shaker-related Kv1 (Kv1.1-Kv1.8) channels display characteristic low threshold voltage activation ranges, well suited to regulate action potential (AP) threshold and waveform, global or local excitability, axon conduction, and synaptic transmission^1–5^, as well as the timing, pattern, and precision with which neurons generate spikes^6–8^. Kv1 channel proteins (except for the Kv1.7 member) are expressed in the brain, as heteromeric, but also homomeric assembly of four Kv1-alpha subunits^2^. Diversity in composition, clustering, and location in different cellular compartments supports Kv1 channels varied functional roles^2,4^. The discovery of toxins specific for Kv1 channel subtypes has been crucial to elucidate their function in different brain areas and neurons^9^.

The finding of point mutations of the gene KCNA1 (Kv1.1) associated to a familiar disorder characterized by attacks of ataxia with or without myokimia, episodic ataxia type-1 (EAT-1)^10^, indicated a critical role for Kv1.1 channels in cerebellar function. Later, it was found that mutations in the KCNA2 gene (Kv1.2) may result in mice^11^ and humans in ataxia and convulsions^12–14^. Kv1 channels, in particular, Kv1.1, 1.2 and Kv1.6 are highly expressed in the cerebellar cortex, and in particular Kv1.1 and Kv1.2 channels are highly expressed in the terminals of the inhibitory basket-cells of the cerebellar cortex^15,16^. Consistently, application of a specific Kv1 channel blocker, dendrotoxin-alpha (DTX_alpha)^9^, greatly enhanced the amplitude and frequency of spontaneous inhibitory postsynaptic potentials (IPSCs) in Purkinje cells, likely mediated by the block of a fraction of the basket-cell terminal potassium outward currents^17^. Furthermore the frequency and amplitude of spontaneous IPSCs in Purkinje cells is found enhanced in a mouse model of EAT-1^18^. Therefore, aberrant evoked synaptic transmission at the basket-Purkinje cell synapses is a likely major cause of the ataxic symptoms of EAT-1^4^. However, since Kv1, particularly heteromeric Kv1.1 and Kv1.2 channels are also expressed at the output cerebellar stage by the deep cerebellar nuclei neurons (DCNs), it has been suggested that aberrant DCNs Kv1 channels could contribute to the EAT-1 cerebellar symptoms, supported by findings that glutamatergic principal DCNs activity is sensitive to specific Kv1 channel blockers^19^. In addition to the principal DCNs, the cerebellar nuclei are composed by diverse groups of GABAergic neurons which are assumed to play important roles in cerebellar function but it remains unclear whether and how their function is regulated by Kv1 channels, as is the case for the principal DCNs. The GABAergic DCNs include: first, the group of small GABAergic neurons forming the nucleo-olivary pathway, which is thought that by directly inhibiting and/or regulating the electrical coupling of inferior-olive cells control the spatio-temporal pattern and probability of cerebellar complex-spikes occurrences and therefore motor control and learning^20–25^. Second, a group of local interneurons co-releasing GABA and glycine that innervates the principal DCNs and could, therefore, directly modulate the cerebellar output^26–28^. And, third, a group of medium sized GABAergic/glycinergic cells, mostly silent in slices in contrast to all other DCNs, which provides a feedback inhibitory pathway to the cerebellar cortex Golgi cells, potentially regulating activity at the cerebellar cortex input stage^28,29^. Here, using cerebellar slices from GAD67-GFP mice line^30^ to identify putative GABAergic-DCNs and a pharmacological approach we found evidence that indeed, the activity of GABAergic-DCNs is regulated by Kv1 channels and thus their alteration could contribute to the symptomatology of EAT-1.

## Results

Here, to elucidate whether Kv1 channels control the activity of GABAergic DCNs, we used cerebellar slices from the mature (>P21) GAD67-GFP mice^31^, their wild type littermates, or mice with the same background to obtain whole cell current clamp recordings of DCNs from the lateral or interpositus nuclei, before and during application of pharmacological blockers of Kv1 channels (see later for details), under conditions alike to those found *in situ* (see Methods). We identified three classes of DCNs according to the presence of fluorescence, size, and electrophysiology (for details see Methods): 1) putatively GABAergic DCNs^31^ (GAD + DCNs), 2) putatively glutamatergic principal DCNs^31^ and, 3) putative nucleo-cortical GABAergic/Glycinergic DCNs^26,28^.

### The GAD + DCNs somatic action potential waveform is sensitive to α-DTX (DTX-alpha)

**Figure 1:**
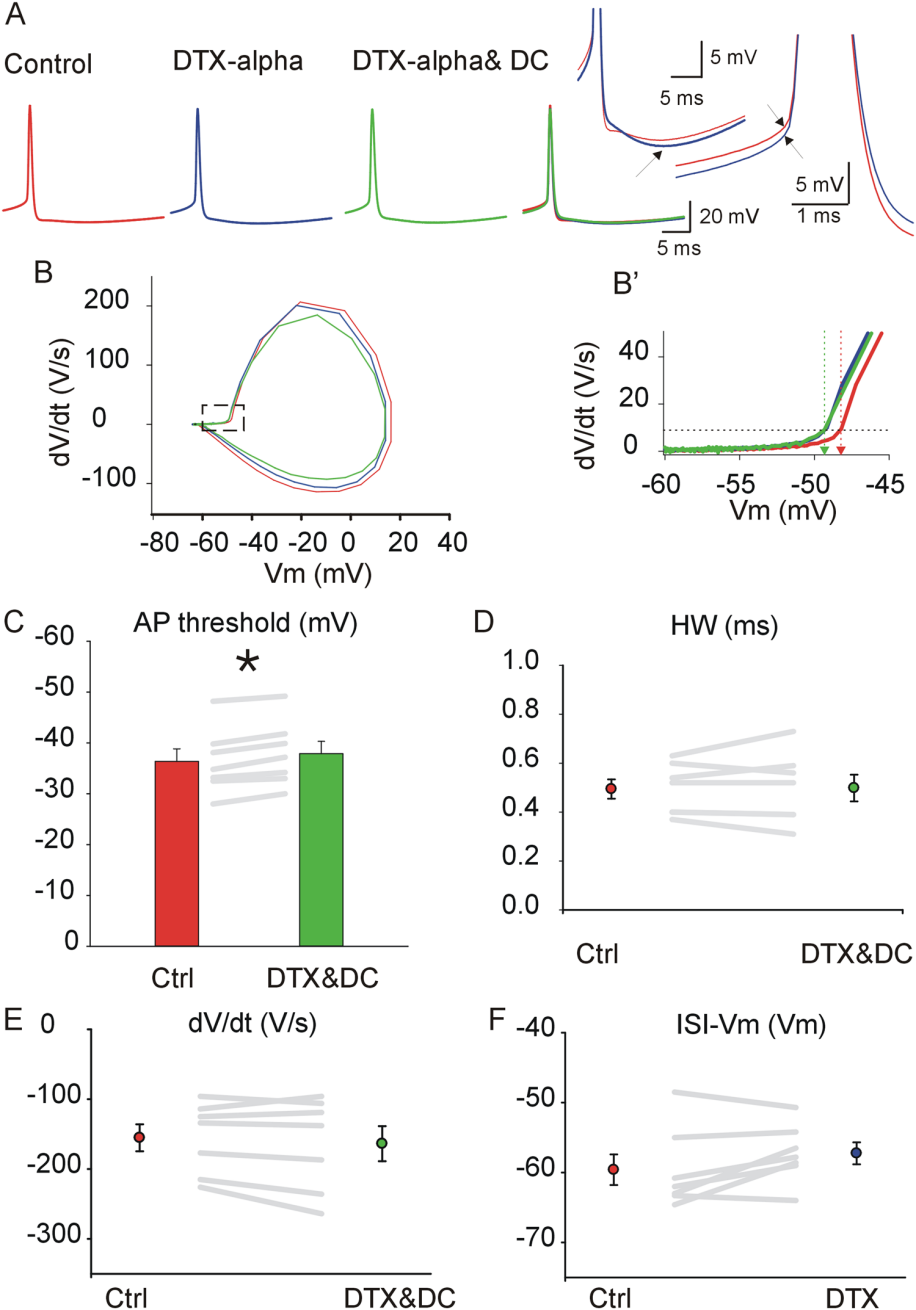
The action potential (AP) waveform of GAD + DCNs is sensitive to DTX-alpha application. (**A**) From left to right: Typical example of averaged spontaneous APs recorded from one GAD + DCN during application of neurotransmitter blockers (control), during DTX-alpha (100 nM) application (DTX-alpha), and after correcting the ISI Vm by current injection during DTX-alpha application (DTX-alpha& DC), and all traces superimposed. The insets depict in expanded scale the control and DTX-alpha traces. The first one illustrates the hyperpolarization during the ISI (marked by the arrow). The second one, the different voltage threshold (noted by arrows). (**B**) Phase plots corresponding to the AP traces in A (the derivative of the Vm as a function of the Vm). B’-Detail of B in expanded scale showing the shift in AP voltage threshold (indicated by the horizontal dashed line, see methods) from control (red vertical dashed line) to more hyperpolarized levels during DTX, even after matching the ISI-Vm (green vertical dashed line). (**C**) Mean AP threshold for all recorded GAD + DCNs under control (red bar) and DTX-alpha application (ISI-Vm matching control levels, green bar) and individual results (depicted by the gray lines). Error lines indicate here and in the next figures SEM. (**D**) Mean AP HW for all recorded GAD + DCNs under control (red dot) and DTX-alpha plus DC (green dot), with individual results depicted as in (**C**). (**E**) Mean AP repolarizing rate (minimum dV/dt) for all recorded GAD + DCNs under control (red) and DTX-alpha plus DC (green), with individual results depicted as in (**C**). (**F**) Mean minimum ISI-membrane potential (ISI-Vm) for all recorded GAD + DCNs under control (red) and DTX-alpha plus DC (green), with individual results as in (**C**).

**Figure 2:**
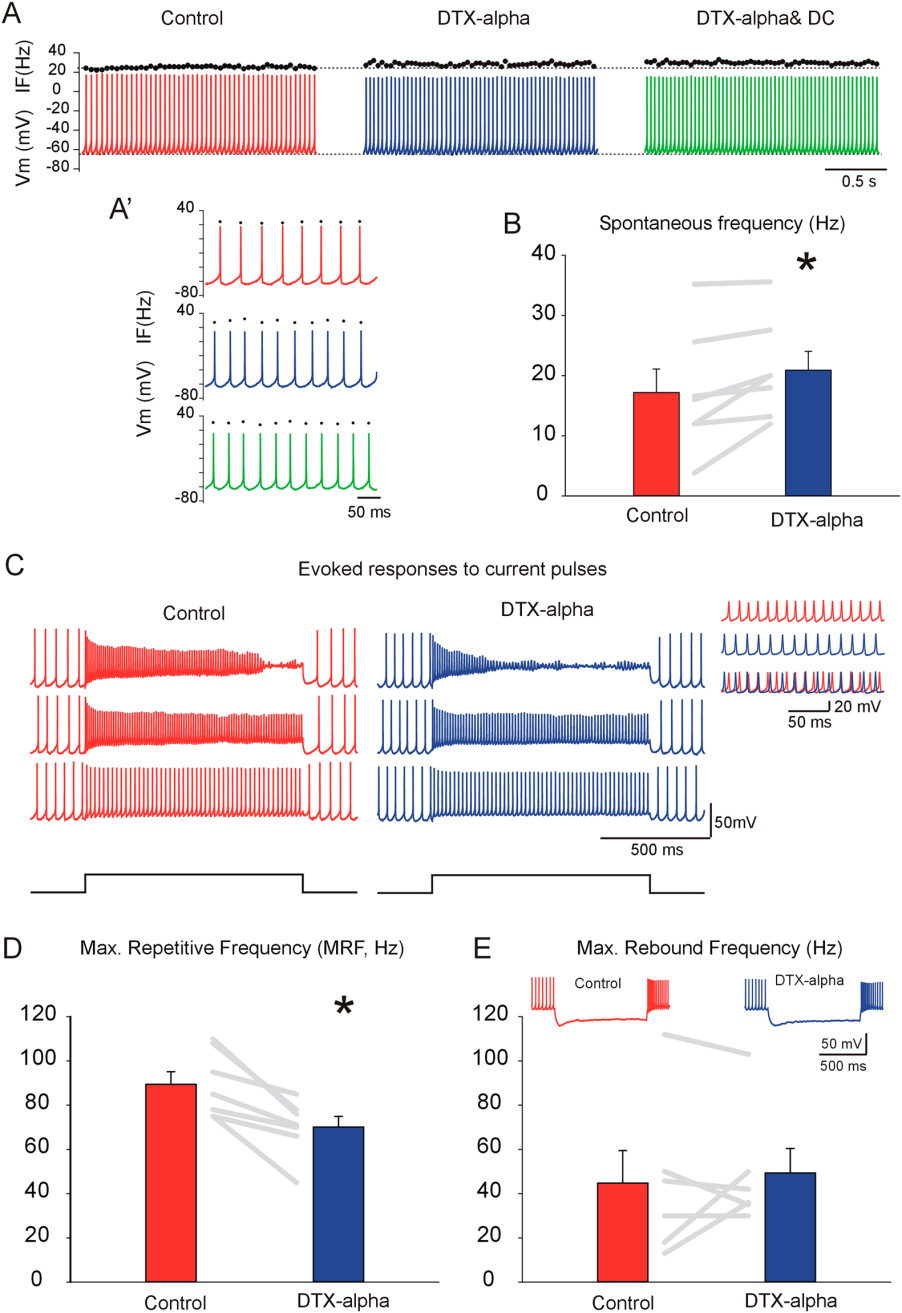
The spontaneous and evoked changes in AP firing activity of GAD + DCNs are sensitive to DTX-alpha. (**A**) From left to right: The traces illustrate typical example of spontaneous action potentials recorded from one GAD + DCN under control conditions (red), DTX-alpha (100 nM, DTX-alpha, blue), and with current injection to match the control ISI-Vm during DTX-alpha application (DTX-alpha& DC, green see text for details). (For comparison, same neuron as in Fig. 1A). The black circles on top of each spike represent the instantaneous frequency (IF, Hz). Note the scale on the left indicate both the membrane potential (Vm) and the IF. A’ detail of A in expanded time scale. The color code used here applies to all figures. (**B**) Bar plots of the mean spontaneous frequency under control and during DTX-alpha application and changes in individual results indicated by the gray lines (see details in main text). (**C**) Top, left: typical examples from the same GAD + DCNs illustrating changes in spiking activity induced by injection of depolarizing current pulses of 1 second duration and increasing current intensity (bottom to top), used to determine the maximum repetitive frequency (MRF), defined as the maximum frequency at steady state before depolarizing block (middle trace, detail shown in the inset) (see methods for further details). (**D**) Mean MRF for GAD + DCNs before (control) and during DTX-alpha application, individual results depicted as in B (details in the main text). (**E**) Mean maximum rebound frequency, the firing rate over the first half second after the step (as in the examples in the inset, see methods for details, “maximum rebound frequency under control (red) and during DTX application (blue), and individual results depicted as in (**B**) (see main text for details).

In a first series of experiments we analyzed whether α-dendrotoxin derived from the Dendroaspis angusticeps (DTX-alpha, 100 nM), which blocks with low EC_50_ channels containing at least one Kv1.1, Kv1.2 or Kv1.6 subunit^2,9^, modulates GAD + DCN spontaneous action potentials (APs). The toxins were applied under the presence of neurotransmitter blockers (see methods), to prevent indirect effects due to changes in the spontaneous release of excitatory or inhibitory neurotransmitters (Figs. 1 and 2). Because DTX application often induced changes in the inter-spike membrane potential (ISI-Vm, see later, and first inset Fig. 1A), and it is known that, at least in putative principal DCNs, the AP waveform is very sensitive to changes in the ISI-Vm^32,33^, constant current injection was applied during DTX application to obtain APs recordings with ISI-Vm matching the control levels for comparison (Fig. 1A, see methods; Control is depicted in red, DTX in blue, and DTX plus DC to match ISI-Vm in green in this, and all the next figures). Because single APs exhibit variable waveform depending on the preceding ISI-Vm, the waveform analysis was carried on averaged APs (≥50 APs) with matching ISI-Vm (see methods for details) and their corresponding phase plots (i.e., the time derivative of the membrane potential as a function of the membrane potential, Fig. 1B and B’, see methods for details) as previously^33^. The most consistent DTX-alpha effect was hyperpolarization of the voltage threshold of spontaneous APs, as illustrated in the example of Fig. 1A, second inset, B and B’(from −48.2 to −49.2 mV, red to green arrows in Fig. 1B’). The threshold hyperpolarized in all 7 neurons investigated and in average the change in threshold was −1.53 mV ± 0.26 (Wilcoxon signed rank test (WSRT), P = 0.016, n = 7, Fig. 1C). Interestingly, the hyperpolarizing shift in voltage threshold occurred despite associated increases in spontaneous firing rate which can be associated to depolarizing shifts in AP threshold^34^, suggesting a strong control by Kv1 channels of this parameter, as will be discussed later.

Kv1 channels have been implicated in some neurons, e.g. neocortical cells, in the repolarizing phase of action potentials^35^. In GAD + DCNs the AP duration, estimated by the AP width at half amplitude (HW), was increased in some neurons but decreases in others during DTX-alpha application and as a population no significant trend was detected (Fig. 1D, WSRT, n = 7, P = 1). To further investigate if DTX modified specifically the repolarizing phase of APs, we analyzed the repolarizing rate (estimated by the minimum dV/dt), however, increases and decreases were found amongst different neurons and no significant change could be detected at the population level (Fig. 1E, WSRT, n = 7 P = 0.297).

Analysis of the ISI-Vm before and during DTX-alpha application (measured without current injection) showed depolarization or hyperpolarization in different neurons, with five out of seven displaying depolarization (Fig. 1F, WSRT, n = 7, P = 0.109).

Overall, these results suggest that DTX-alpha sensitive channels are present in GAD + DCNs and regulate the AP voltage threshold, while other waveform parameters are modulated by the same channels but the effect varies amongst different neurons. The implications will be discussed later.

### GAD + DCNs AP firing activity is sensitive to DTX-alpha

Next, we investigated the sensitivity of AP spontaneous and evoked firing activity to DTX-alpha. Application of DTX-alpha led to consistent increases in the spontaneous firing frequency as in the example of Fig. 2A and detailed in 2A’ (color code as in Fig. 1), where the spontaneous mean rate increased from 25.6 Hz under control conditions to 27.6 Hz under DTX application. Increases in spontaneous rate were found in all neurons tested (WSRT, n = 7, P = 0.016, Fig. 2B), on average by 3.7 ± 1.33 Hz. This outcome was independent of whether DTX-alpha application induced depolarization or hyperpolarization of the ISI-Vm. For instance, although in the example of Fig. 2A the ISI-Vm hyperpolarized (same example as in Fig. 1), the spontaneous firing rate increased (depicted by the black circles on top of the spikes traces Fig. 2A, A’). Furthermore, constant (depolarizing) current injection to match ISI-Vm to control levels resulted in even further increase in firing rate to 29.4 Hz in this case (Fig. 2A, rightmost plot and 2A’. bottom plot).

DTX-alpha interfered with the ability of GAD + DCNs to fire repetitively at high frequency, estimated by the maximum steady state repetitive frequency evoked by depolarizing current pulses of one second duration and increasing intensity (Fig. 2C, steady state activity detail in inset, MRF, see methods for details). The MRF significantly decreased during DTX-alpha application (Fig. 2D, WSRT, n = 7, P = 0.016). This suggests that a Kv1 outward current is necessary for sustaining high frequency firing, presumably by enabling sufficient sodium channel de-inactivation between the spikes.

Large principal and GABAergic DCNs usually respond with an increase in firing rate above baseline after the end of hyperpolarizing stimuli (rebound response) e.g.^31,36^, as illustrated in the example of the inset of Fig. 2E. DTX induced changes in the maximum rebound frequency (see methods for details), but both increases and decreases were detected in different neurons and no statistical significant trend was detected (Fig. 2E, WSRT, n = 6, P = 0.81).

Taken together these results strongly suggest that in GAD + DCNs express functional Kv1 channels that are sensitive to DTX-alpha, and that these channels have a role in limiting GAD + DCNs spontaneous activity while facilitating high frequency responses to excitatory inputs.

### GAD + DCNs are also sensitive to DTX-I and DTX-K

We tested the effect of two other polypeptides isolated from the Dendroaspis polylepis venom (Dendrotoxin-I (n = 7) and dendrotoxin-K (n = 6), both 100 nM), both blocking Kv1.1 and Kv1.2 channels, althoughDTX-K may have higher selectively for channels formed by Kv1.1 subunits. After DTX-K application depolarizing block was observed in two neurons, and therefore these neurons were not included in the analysis^9^. Regarding the effect on the spontaneous APs, we found that that these two toxins induced hyperpolarization of the AP voltage threshold (measured as before on averaged APs with ISI potential matched to control levels) in all tested neurons but one, which showed no difference before and during DTX-I application. The action potential duration (HW) was slightly increased in all tested neurons, except one where a decrease in HW was found (using DTX-K). Although DTXs induced changes in the ISI-Vm, these occurred in different directions in different neurons and were not correlated to the toxin used.

Regarding the spontaneous AP firing rate we found increases in most neurons tested (4 out of 4 neurons with DTX-K and 5 out of 7 with DTX-I). Furthermore, application of DTX-K/I led to decreases in the MRF (in 8 out of 10 neurons tested). Regarding the hyperpolarization triggered maximum rebound frequency we found diverging effects as with DTX-alpha. Summarizing, we found similar results using the three different toxins. Because DTX-K is proposed to be more specific for Kv1.1 channels, the similarity of results using different DTXs supports the idea that the population of channels expressed by GAD + DCN are heteromeric and contain at least one Kv1.1 subunit^9,19^.

Given the similarity of results using the different toxins, next, we decided to pool all results together to further explore in the whole population the functions of Kv1 channels.

### Kv1 channels regulate the spiking activity and membrane potential of silent and spontaneously active GAD + DCNs

**Figure 3:**
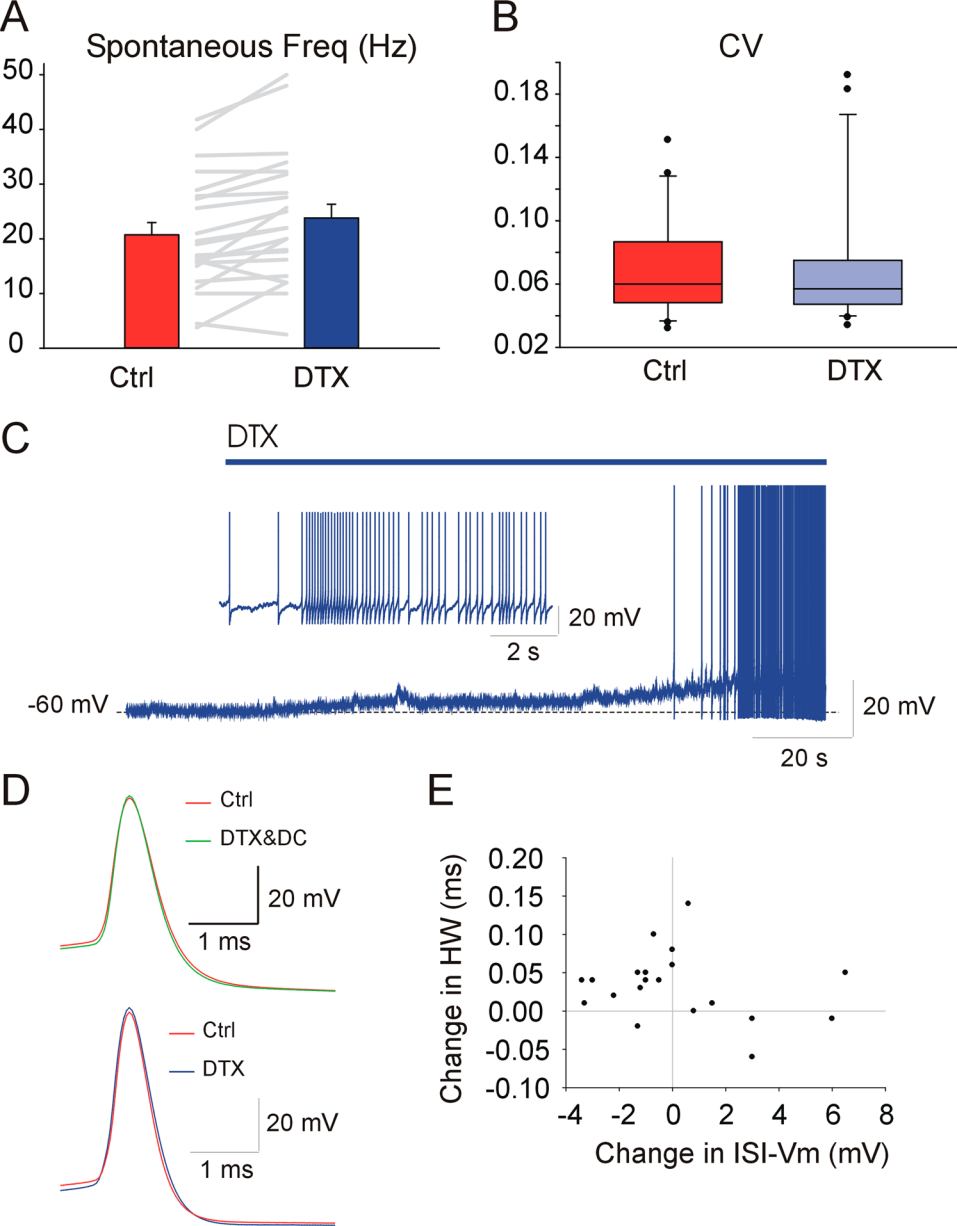
Kv1 channels control the spiking activity and membrane potential of silent and spontaneously active GAD + DCNs. (**A**) Mean spontaneous frequency under control and DTX conditions (data from DTX-alpha, DTX-I and DTX-K together, no DC applied, see details in main text). (**B**) CV of spontaneous ISIs under control and DTX conditions (box illustrates 10 and 90% percentiles and whiskers the error, see details in main text). (**C**) DTX effect on a previously silent DCN (DTX-alpha, 100 nM, time of application indicated by the bar on top), illustrates the depolarization and beginning of tonic firing evoked by DTX. The inset illustrates in expanded time base the beginning of the tonic discharge. (For clarity the responses to short pulses (10 ms) applied every two seconds before spiking started were digitally removed and substituted by a straight line). (**D**) Two examples of GAD + DCN recorded using 25 KHz sampling rate, illustrate typical DTX effects on averaged spontaneous APs. Top, in this example DTX induced a depolarization of the ISI-Vm. After DC injection to match control ISI-Vm (green trace), the spike width was narrower than control (HW: 0.59 and 0.56 for control and DTX&DC respectively). Bottom, in this example, the ISI-Vm hyperpolarized and the AP duration increased during DTX (HW: 0.49 and 0.54 ms for control and DTX respectively). (**E**) Scatter plot of DTX induced changes in HW (ms) as a function of the changes induced in ISI-VM (mV, note that most neurons that hyperpolarized displayed elongations in HW).

In line with the results using different DTXs, analysis of the pooled data indicated a significant increase in the spontaneous firing rate, (Fig. 3A, on average from 20.7 ± 2.23 to 23.8 ± 2.5 Hz, WSRT, n = 22, P = <0.001). Previous studies reported that Kv1 channel blockade in principal, putative glutamatergic DCNs, in addition to increasing the firing rate, degraded the rhythmic pacemaker activity, as quantified by changes in the coefficient of variation (CV) of inter-spike-intervals (ISI)^19^. Thus, we explored whether DTX changed the regularity of GAD + DCNs (Fig. 3B). However, in contrast to the results in principal DCNs, no significant changes in the CV of spontaneous ISIs was detected (0.071 ± 0.007 and 0.071 ± 0.009, WSRT, n = 22, P = 0.93). Furthermore, inspection of GAD + DCNs recordings did not reveal irregular firing nor the occurrence of “spikelets” (implicated in the irregular firing in principal DCNs^19^). These results strongly suggest that Kv1 channels control the spontaneous firing rate of GAD + DCNs, but their pacemaker properties do not seem to be affected by Kv1 channel suppression, at least in GAD + DCNs from mature mice.

Next, we explored the question of how Kv1 channels control GAD + DCN spontaneous firing rate. At least two different, non-excluding mechanisms could be responsible for the increases in rate: First, Kv1 channels could control the rate by controlling the AP threshold. In this direction, we found strong evidence that DTXs hyperpolarized the AP threshold. As expected, analysis of the whole population revealed consistent and significant changes in the threshold of spontaneous spikes, which shifted in hyperpolarizing direction during DTXs application (on average, considering all the pooled data, by 1.5 mV, WSRT, n = 18, P < 0.0001). Although our recordings were obtained from the soma of GAD + DCNs, this effect most likely reflects the change in threshold of the AP triggering zone, i.e. the axon initial segment^3^. Thus, by hyperpolarizing the AP threshold, Kv1 channel blockers likely promoted higher spontaneous firing rates.

Second, Kv1 channels could regulate the spontaneous firing rate by maintaining a tonic outward current. Support for this idea was obtained from a series of recordings from a group of non-spontaneously active DCNs. These neurons likely correspond to the group of GAD + DCNs co-expressing glycine and GABA as neurotransmitter, and forming a recurrent nucleo-cortical pathway targeting Golgi cells, which are characteristically silent when recorded from slices, in contrast to all other types of DCNs^26,28^. Application of DTX induced depolarization (on average by 4.1 ± 1.4 mV, n = 5, DTX-alpha or -I, 100 nM), as in the example illustrated in Fig. 3C. In this example, DTX application depolarized the membrane potential and evoked tonic firing (two out of the five silent DCNs became active under DTX). These results are compatible with the idea that Kv1 channels maintain a tonic outward current sufficient to prevent these neurons from reaching AP threshold. Regarding the spontaneously firing GAD + DCNs, application of DTXs depolarized some of them (9 out of 21). The depolarization of these neurons could have resulted from the block of a steady outward Kv1 current, as in the silent DCNs. However, a similar number of neurons hyperpolarized and no significant effect on the ISI-Vm was found analysing the whole population (WSRT, n = 21, P = 0.80). Because silent DCNs exhibited a more hyperpolarized membrane potential than the spontaneously active ones, we first wondered whether the different effects of DTX on the ISI-Vm were dependent on the Vm level before DTX application. However, the correlations between the changes in ISI-Vm and the absolute ISI-Vm before DTX were low (R^2^ = 0.09), suggesting the different effects were not due to the preceding Vm. Overall, these results provide evidence that Kv1 channels tonically hyperpolarize the membrane potential of silent DCNs, and suggest this could be also the case for some spontaneously firing GAD + DCNs, but not others.

The previous results opened the question of why some spontaneously active GAD + DCNs were hyperpolarized by DTX applications. Kv1 channels drive outward currents at the spontaneous DCNs membrane potential, and therefore the hyperpolarization cannot be a direct effect of DTX blocking the Kv1 channels. However, since the ISI-Vm is in part controlled by currents activated by the spontaneous spikes, DTX could have modified spike-dependent currents. In particular, if Kv1 channels contributed to the repolarization of somatic APs, then, DTX could have resulted in increased AP duration and secondary enhancement of calcium and/or voltage dependent outward currents (e.g. SK ones) during the ISI. Indeed, by analysing the pooled data from all DTXs applications together, we found a small but significant increase in AP HW (4.8 ± 2.3%, WSRT, n = 16, P = 0.03). However, the DTX effect in the repolarization rate (estimated by the minimum in dV/dt) was not significant (WSRT, n = 15, P = 0. 65), although the changes in the AP minimum dV/dt were correlated to the changes in the AP HW (i.e. the increases in spikes width were correlated to less negative dV/dt, R^2^ = 0.51). Furthermore, the results were confirmed in a different set of experiments using higher sampling rates (25 KHz) to detect changes in HW with higher sensitivity(DTX-I, 100 nM, the somatic AP threshold hyperpolarized by 1.8 ± 0.59 mV, WSRT, n = 7, P = 0.031; the AP HW exhibited a small but significant increase, of 0.03 ms on average, from 0.5 ± 0.04 to 0.52 ± 0.03 ms, WSRT, n = 7, P = 0.047; and the minimum dV/dt decreased non significantly,WSRT, n = 7, P = 0.56). Two typical examples of GAD + DCN displaying either no change (or decrease) or increase in spike duration under DTX application are illustrated in Fig. 3D, both acquired using 25 kHz sampling rate. One possible interpretation of these results is that Kv1 channels contribute to the AP repolarizing phase, but in different proportion in different neurons, such that in some neurons the direct effect on the ISI predominates and in others the indirect one via the slowed repolarization. In support of this idea we found that although the changes in ISI-Vm were not well correlated to those in AP-HW (R^2^ = 0.05, n = 20), most neurons that hyperpolarized during DTX application displayed as well, increases in the AP HW (Fig. 3E). Furthermore, correlation of the same parameters in the population of GAD + DCNs recorded at higher sampling rate showed a good correlation (R^2^ = 0.68, n = 7). Briefly, DTX hyperpolarized the ISI-VM of some GAD-DCNs, likely by interfering with the AP repolarization.

Finally, regarding the effect of DTX on evoked activity the analysis of the pooled data showed significant decreases in the MRF (WSRT, n = 19, P = < 0.001). In contrast, population analysis of the changes in maximal rebound frequency during DTXs application did not reveal a significant trend (WSRT, n = 19, P = 0.82).

Summarizing, these results point to an important role of Kv1 channels in dampening or controlling the tonic firing rate of spontaneously active and silent GAD + DCNs, by at least two different mechanism, AP voltage threshold and/or membrane potential control. Moreover, the results suggest that Kv1 channels facilitate the high frequency activity evoked by depolarizing inputs.

### The AP waveform and firing activity of putative principal DCNs is sensitive to DTX

**Figure 4:**
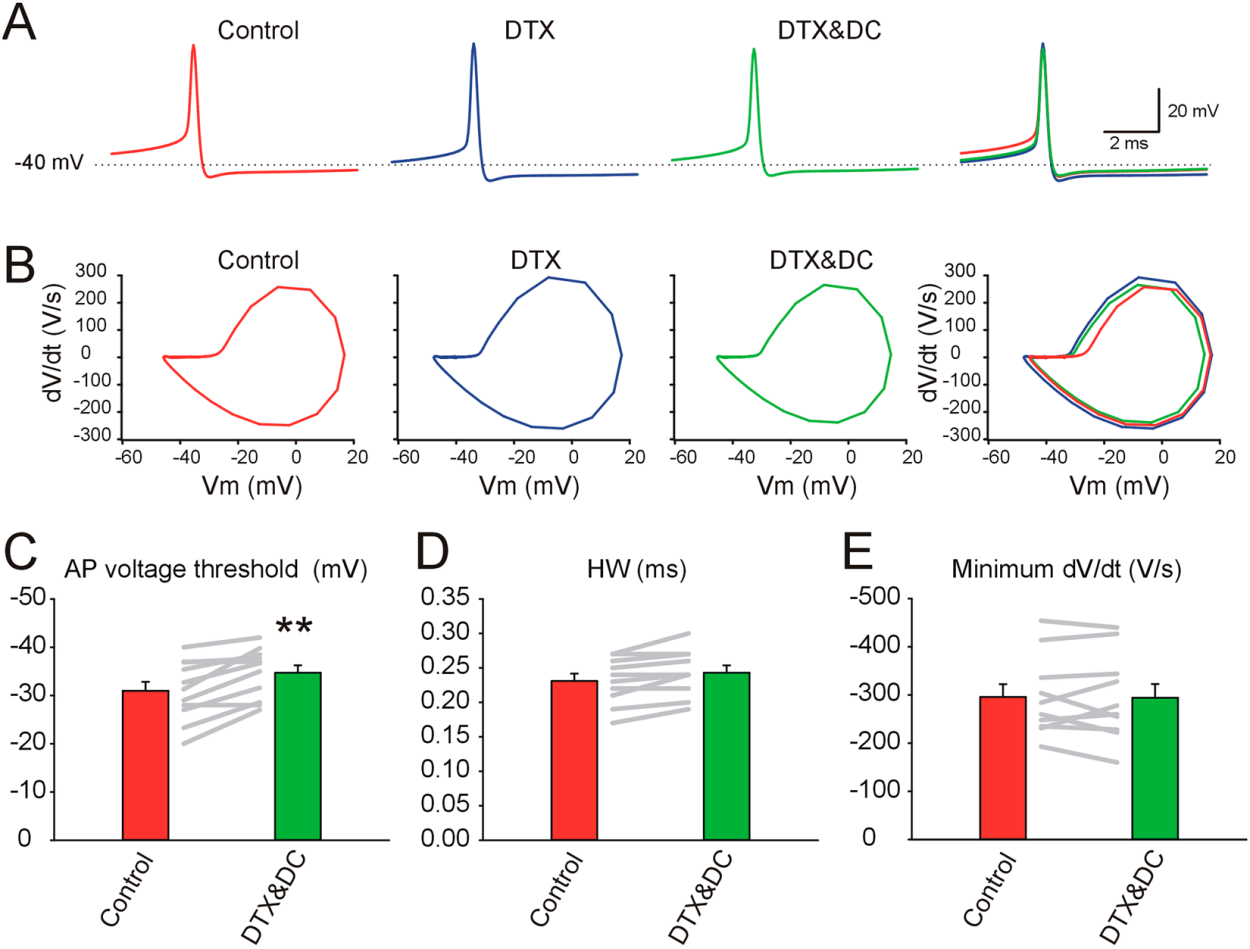
The action potential waveform of non-GAD + DCNs, putative glutamatergic principal DCNs is sensitive to DTX. (**A**) From left to right: Typical example of averaged spontaneous action potentials recorded from a large non-GAD + DCN during application of neurotransmitter blockers (control, red), during DTX-alpha (100 nM) application (DTX-alpha, blue), and after matching the ISI Vm to control levels by current injection during DTX-alpha application (DTX-alpha& DC, green), and all traces superimposed. (**B**) The corresponding phase plots of the traces illustrated in A. (**C**) Mean AP threshold before and during DTX application for putative glutamatergic principal DCNs and individual results depicted by the gray lines (see details in main text). (**D**) Mean AP HW before and during DTX application for putative glutamatergic principal DCNs and individual results depicted as gray lines (see main text for details). (**E**) Mean AP repolarizing rate (minimum dV/dt) before and during DTX application for putative glutamatergic principal DCNs and individual results as gray lines (see details in main text).

**Figure 5:**
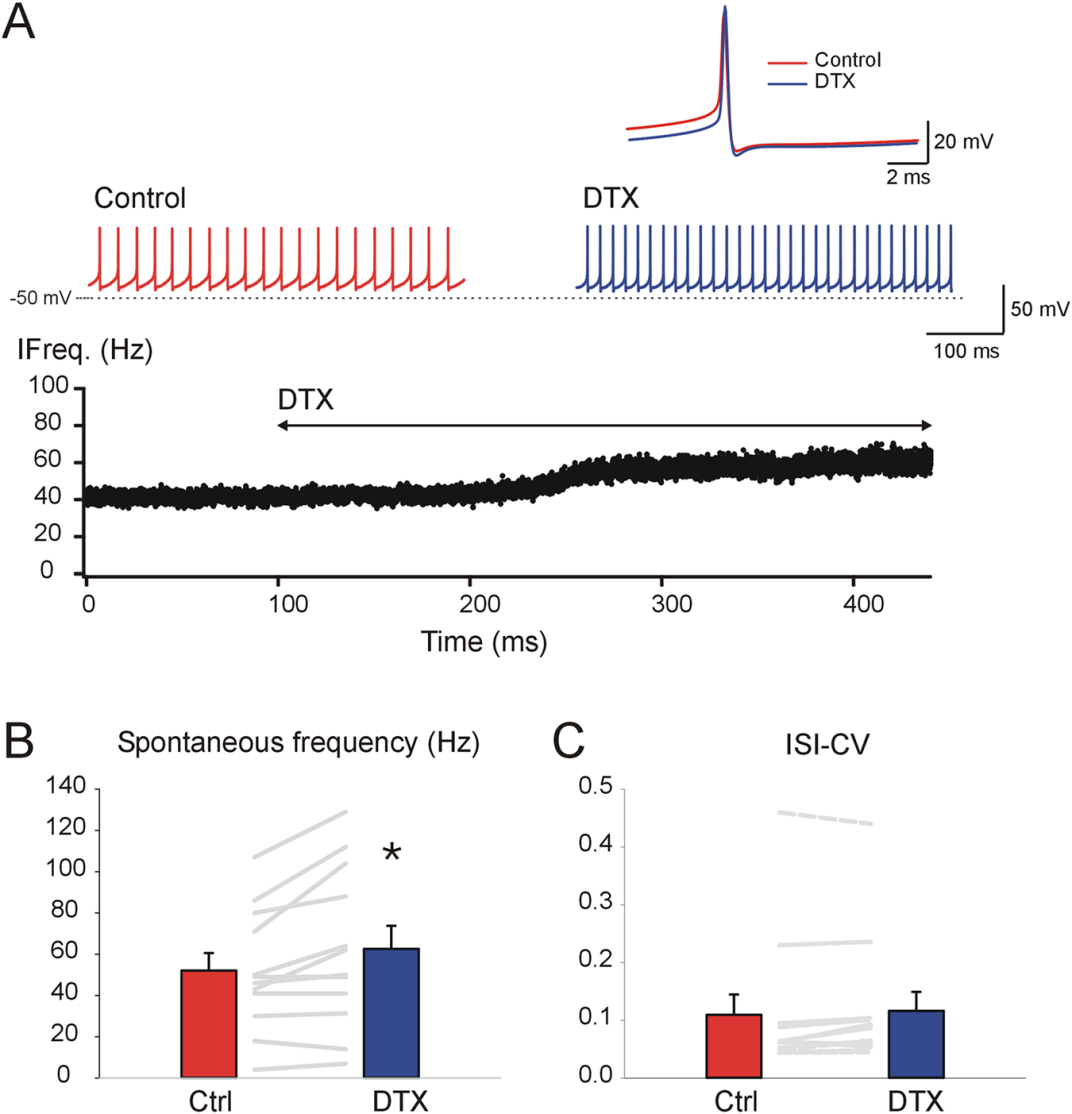
Kv1 channels control the spontaneous firing rate of non-GAD + DCNs, putative glutamatergic principal DCNs. (**A**) The red and blue traces illustrate segments of the whole cell current clamp recordings of a spontaneously active putative glutamatergic principal DCNs before and during application of DTX-I (100 nM) respectively. Note the depolarization and decrease in ISI between APs during DTX application. The inset above shows the averages of APs obtained before and during DTX application (red and blue, respectively). The bottom plot illustrates the changes in instantaneous frequency induced by application of DTX to the bath (indicated by the black line). (**B**) Mean spontaneous frequency before and during DTX application for putative glutamatergic principal DCNs and individual results depicted in gray lines (details in main text). (**C**) Mean CV of the ISIs of spontaneous AP before and during DTX application for putative glutamatergic principal DCNs and individual results (details in main text).

To be able to compare the effect of DTX between different DCN classes here we investigated the effect of DTX on putative glutamatergic principal DCNs using the same recording conditions, i.e., age of mice, temperature, calcium concentration, and type of analysis for GAD + DCNs (Figs. 4 and 5). For this set of experiments we used DTX-alpha and DTX-I, and the results were pooled together for analysis.

Similar to the case with GAD + DCNs, and in agreement with previous results^19^, DTX application modified the activity of putative principal DCNs, as in the example of Fig. 4A and corresponding phase plots in Fig. 4B. Hyperpolarization of the AP voltage threshold was the most consistent effect of DTXs on the AP waveform (on average by 3.7 ± 0.86 mV, WSRT, n = 11, P = 0.004, Fig. 4C). The AP duration estimated by the HW was slightly (on average only 0.01 ± 0.003 ms, Fig. 4D), but significantly increased under DTX application (WSRT, n = 10, P = 0.016). However, no significantly changes in the repolarizing rate were detected (WSRT, n = 10, P = 0.92, Fig. 4E). The ISI membrane potential depolarized or hyperpolarized in different neurons similar to the effect of DTX on GAD + DCNs (WSRT, n = 11, P = 0.74). Furthermore, similar results arose when we investigated the effect of DTX on a group of large DCNs recorded using higher sampling rate to improve the detection of small changes in AP waveform: the threshold hyperpolarized (on average by 3.9 ± 0.81 mV, WSRT, n = 7, P = 0.031). The HW changed in different directions and no significant trend was observed (from 0.22 ± 0.02 to 0.23 ± 0.02, WSRT, n = 7, P = 0.156). Similarly, no significant differences were detected in the AP repolarization rate (estimated by the minimum dV/dt, WSRT, n = 7, P = 0.375).

Regarding the effect of DTX on the AP firing activity, DTX led in most neurons to increases in spontaneous firing rate (Fig. 5A,B, WSRT, n = 12, P = 0.01)) in agreement with previous results^19^, on average by 20 ± 0.07% respect to corresponding control levels. We did not detect significant changes in the coefficient of variation (CV) of the inter-spike intervals (ISI) during DTXs application (WSRT, n = 12, P = 0.110, Fig. 5C), suggesting that under the present recording conditions DTXs application did not interfere with the regularity of the putative principal DCNs pacemaker activity. Furthermore, in the present study we did not detect the presence of “spikelets” before or during DTX application in recordings from putative principal DCNs (e.g. Fig. 5A).

DTX application decreased the ability of putative principal DCNs to fire repetitively at high frequencies in response to the injection of depolarizing current pulses (estimated by the MRF, 471 ± 33.4 and 412 ± 28 Hz for control and DTX respectively, WSRT, P = 0.023 n = 9), in agreement with previous results^19^. On the other hand, DTX application significantly increased the maximum rebound frequency induced after the injection of anodic current pulses (189 ± 34 and 220 ± 41 Hz for control and DTXs respectively, WSRT, n = 11, P = 0.024), also in agreement with previous results^19^.

Summarizing, DTX application shifted the AP threshold to more hyperpolarized levels, increased the spontaneous firing rate, decreased the ability to fire at high frequencies and increased the frequency of rebound responses elicited at the end of hyperpolarizing stimuli of putative principal DCNs.

## Discussion

The present results indicate that the activity of putative GABAergic GAD + DCNs is sensitive to DTX-alpha, DTX-I and DTX-K. In particular, all DTXs shifted the voltage threshold of spontaneous AP recorded in the soma of GAD + DCNs in the hyperpolarizing direction, increased the spontaneous firing rate and decreased the ability to fire at high frequencies in response to depolarizing inputs of GAD + DCNs. Moreover, in a group of previously silent DCNs, putative nucleo-cortical inhibitory DCNs, application of Kv1 channel blockers induced depolarization and in some cases tonic firing of action potentials for the duration of the blocker application. Furthermore, we confirmed that putative principal glutamatergic DCNs are sensitive to DTXs application. Overall, these results strongly suggest that Kv1 channels are expressed by GABAergic DCNs, and argue for a role of Kv1 channels in stabilizing the AP threshold, dampening the pacemaker activity of the spontaneously active GABAergic-DCNs, while facilitating their ability to respond with high frequency to depolarizing inputs.

Kv1 channels display typical low voltage activation ranges and therefore are well suited to control the AP threshold and neurons excitability^2,3,35,37,38^. The AP voltage threshold is assumed to result from the interplay between the inward sodium current and the outward currents active around the point in space and time where the sodium current becomes regenerative^1^. DTX shifted in the hyperpolarizing directions the voltage threshold of GAD + DCN spontaneous APs, strongly suggesting that Kv1 outward currents normally counteract the inward currents responsible for driving the spikes which then could prevail at more hyperpolarized levels. The shift in threshold is probably a major cause for the increase in spontaneous frequency during DTXs application, as increased frequency was observed in neurons displaying either hyper or depolarization of the inter-spike membrane potential (e.g. Fig. 1A). The increase in frequency, by limiting the time available between spikes for sodium channels de-inactivation^32,33^ could have shifted the voltage threshold in the depolarizing direction^34^ and counteracted the effect of DTX, however, the threshold shifted in the hyperpolarizing direction during DTXs application despite increased spontaneous frequency, suggesting a major role of Kv1 currents in regulating the GAD + DCNs AP voltage threshold. In other neurons it has been demonstrated that local clustering of Kv1 channels around the locus of AP initiation, i.e. usually the axon initial segment, is critical for their control of the voltage spike threshold^3^. This possibility could explain how the threshold could have shifted in the hyperpolarizing direction in most neurons (suggesting less outward currents to balance the inward ones), while the inter-spike membrane potential hyperpolarized in some of them during DTX application, suggestive of larger outward currents in the somatic compartment during this phase (discussed later), warranting further studies to explore this idea.

Kv1 channels are involved in the repolarization of somatic APs in some neurons but not others, and may depend on the developmental state^35,37^. We found that DTXs exerted diverging effects on the repolarization rate or the duration of somatic APs, suggesting Kv1 channels do not play a major role on the somatic AP repolarization of GAD + DCNs. However without playing a major role, different balance between direct and indirect effects of Kv1 channels could have resulted in diverging effects in different neurons during DTX application. For instance, block of Kv1 channels contributing to the AP repolarization could directly slow down AP repolarization rate. In contrast, DTX hyperpolarization of AP threshold could lead to increased AP amplitude and indirectly larger activation of AP triggered potassium currents, which could counteract the direct effect of Kv1 channel blockade. Nevertheless in principal DCNs, use of very low doses of 4-aminopyridine, which blocks Kv3.1 channels, results in large changes in the AP duration and repolarization rate^33^, suggesting that DTX sensitive Kv1 channels play only a minor role in principal DCNs AP repolarization, at least for the somatic APs.

Similarly, we found diverse effects of DTXs on the ISI-Vm, with some GAD + DCNs depolarizing and other hyperpolarizing. The depolarization may be the direct result of DTX blockade of a putative tonic Kv1 current present during the ISI, a possibility congruent with non-inactivating character of some of these currents^2,4^. This idea is largely supported by the evidence that DTX depolarized all tested silent, putative nucleo-cortical gylcinergic DCNs (Fig. 3). On the other hand, as the ISI-Vm in DCNs is likely dynamically controlled by diverse channels, some active at this membrane potential and others activated by the spontaneous spikes^32,33,39–41^, DTX could have influenced the ISI-VM indirectly by interfering with currents evoked during the AP, e.g. calcium activated potassium currents, causing an hyperpolarization.

We consider at least two, not excluding causes to explain the diversity of DTX effects: First, GAD + DCNs are a heterogeneous population and thus the type, density or location of membrane channels amongst GAD + DCN classes could be variable and explain the differences found in the present study. A second alternative is that each DCN expresses a range of different proportion (or density or location) of membrane channels, independently of the DCN class, possibly dependent on the type of afferents, type of targets, history, etc., as proposed for other systems^42^. In either case, as similar electrophysiological patterns can be implemented by different ionic channel sets, it could be that the effect of DTX revealed differences not obvious under control conditions^42^.

Our results indicate that the spontaneous firing rate of DCNs is sensitive to Kv1 channel blockers suggesting that Kv1 channels continuously control the output of GABAergic (as well as non-GABAergic) DCNs. In the present study the increase in GAD + DCN spontaneous firing rate was not associated to increased irregularity of firing as has been described before for putative principal DCNs (Fig. 3B)^19^. Surprisingly, we found that under our recordings conditions, DTX application did not induce irregular spontaneous firing in putative principal DCNs, although we confirmed other effects of DTX in putative principal DCNs, i.e. increased spontaneous firing rate, decreased ability to fire at high frequency and enhanced rebound response firing rate. Moreover, we did not observe in our recordings the presence of “spikelets”, which were postulated to originate in axonal spikes re-invading the somato-dendritic compartment and the cause of irregular firing^19^, neither in GAD + DCNs nor in putative principal DCNs. The previous study describing “spikelets” under Kv1 blockade were carried out in slices from animals younger than the ones used here^19^, and could indicate that Kv1 control of “spikelet” activity is developmentally regulated, as proposed for other functions and neurons^37^. In addition, our result do not rule out further roles of Kv1 channels in controlling DCNs AP waveform and transmission through the axon, collaterals, and/or terminals, where may influence synaptic transmission as shown earlier for other cells^5,43,44^.

Overall, the present results strongly suggest that Kv1 channels in GABAergic DCNs regulate the threshold of somatically recorded APs, dampen the basal firing rate and facilitate high frequency responses to depolarizing inputs. These effects could serve to enhance the saliency of excitatory inputs to GAD + DCNs. By regulating the spontaneous and evoked firing rate of GABAergic DCNs, Kv1 channels could influence 1) The occurrence and spatiotemporal patterns of Purkinje cells complex spikes by controlling the inhibitory nucleo-olivary pathway activity. The regulation of this pathway could therefore influence cerebellar motor control and learning^20–25^. 2) The cerebellar output by controlling the activity of the locally projecting GABAergic DCNs which innervate the principal glutamatergic DCNs. 3) Kv1 channels could regulate the silent or active state of the nucleo-cortical DCNs^26^. This finding could explain our previous results showing that application of 4-AP (a non-specific potassium channel blockers, which blocks Kv1 channels with low affinity) resulted in large increases in “spontaneous” glycinergic IPSCs recorded from large putative principal DCNs^27^, since the putative nucleo-cortical GAD + DCNs, in addition to the axon projecting to the cerebellar cortex give rise to intra-nuclear collaterals^28^. In addition, Kv1 channels by regulating the tonic level of activity of the glycinergic nucleo-cortical neurons could influence the information processing at the cerebellar input stage^26^.

Taken together these results indicate that spontaneous activity and responsiveness of GABAergic DCNs is controlled by Kv1 channels. From a functional point of view, and since there is evidence that Kv1 channel expression can be modulated by synaptic activity^45^, it will be interesting to investigate the possibility of activity dependent regulation of Kv1 channels in GABAergic DCNs and whether this modulates cerebellar function. From a pathophysiological point of view, these results point to a new cellular basis for cerebellar dysfunction in Kv1 channelopathies, like EAT-1.

## Methods

The animal protocol was reviewed and approved by an independent local committee and the Regional Council of Tübingen and conducted according to the standards of German law and the Society for Neurosciences (SFN).

### Cerebellar slices preparation

For preparing cerebellar slices mice from the GAD67-GFP knock-in mice line (with one GFP protein gene substitutes one GAD-67 allele)^30^ their wild type littermates or wild type animal with the same background (both sexes, >P21, ranging from P21 to P35) deeply anesthetized with ketamine (150 mg/kg) or flurazepan, were prepared as previously^46^ using a vibrotome (Leica, Bensheim, Germany), and ACSF (containing in mM: 125.5 NaCl, 2.5 KCl, 1.3 NaH_2_PO_4_, 1 MgCl_2_, 26 NaHCO_3_, 20 Glucose, 1.5 CaCl_2_), bubbled with 95% O_2_ and 5% CO_2,_ and maintained at 26 °C during preparation. Slices were immediately transferred to a storing chamber with ACSF at 36 °C and afterwards let to cool down to room temperature in the same solution until recorded. The same solution at close to physiological temperatures (33–35 °C) was used for recordings using a submerged type chamber.

### Whole cell Patch clamp recordings

To achieve conditions close to those found *in situ* the slices were maintained during the recordings at close to physiological temperature (33–35 °C) and were perfused with ACSF containing physiological extracellular calcium concentrations (1.5 mM, see above). Whole-cell current clamp recordings were made from DCNs in the lateral or interpositus nuclei using an Axoclamp2B-amplifier (Molecular Devices). DCNs were visualized using infrared illumination and the presence of fluorescence was tested using LED illumination (470 nm, CoolLED). The presence of GFP fluorescence was used to identify putatively GABAergic DCNs^31^ (GAD + DCNs). Non-GFP fluorescent medium sized DCNs (longest diameter >18 µM) recorded from the GAD-GFP mice line slices, and large DCNs (diameter >21 µm) from any mouse line displaying spontaneous spiking activity were identified as putatively glutamatergic principal DCNs^31^. Silent DCNs were identified as putative nucleo-cortical GABA/Glycine DCNs. The intracellular solution contained (in mM): 134 Kgluconate, 6 KCl, 10 KHEPES; 0.1 EGTA, 0.3 NaGTP, 2 KATP, 10 Phosphocreatine, 2 MgCl_2_. Recordings were digitized (12.5 or 25 kHz), and stored using programmable software (Spike 2, CED) for further analysis.

### Drugs

The following drugs: gabazine (3 µM), an antagonist of GABAA receptors; strychnine (1 µM), an antagonist of glycine receptors; kynurenic acid (3–5 mM), a broad-spectrum antagonist of ionotropic glutamate receptors were systematically applied to the ACSF to be able to isolate pharmacologically the effect of the Kv1 blockers on the intrinsic properties of DCNs (Control conditions). (All these antagonists were purchased from Sigma). Kv1 channel blockers were applied to the bath by adding them to the sub-perfusing solution containing the neurotransmitter antagonists and the effect was analyzed after approximately 10 minutes to reach steady state level. The specific Kv1 channel blockers: αDendrotoxin (DTX-alpha), DTX-I and DTX-K (100 nM) were diluted as recommend by the providers (Alomone, Latoxan) to prepare aliquots that were frozen and diluted to the final concentration in ACSF containing neurotransmitter blockers before use. We noticed after analysing the data that the potency of the toxins decreased with time after aliquot preparation. Therefore, when this information was available we excluded experiments using aliquots older than 4 weeks.

### Experimental protocols and analysis

Protocols and analysis were done as previously^33,47^. Briefly: the recordings were performed at the spontaneous membrane potential unless otherwise noted. Only neurons with stable recordings were included for analysis. Rarely, during recordings some neurons “spontaneously” depolarized and remained in depolarized state (depolarizing block). These neurons were not included in the analysis although sometimes they recover. Because one effect of the drug application could be to facilitate this process, we report the occurrences after toxin application, but we did not include data from these neurons in the analysis. For the spike waveform analysis of spontaneous action potentials (APs) we used averages of successive spikes aligned by their peak (≥ 50 APs), obtained under control conditions and after the effect of toxins application stabilized (usually 10 minutes). Because the fraction of sodium current inactivated at the spontaneous DCNs membrane potential is high^32^, the spike waveform is very sensitive to changes in ISI-Vm as shown earlier^33^. For this reason, after obtaining basal recordings under Kv1 blockers, the bridge balance was monitored and constant current was injected to repolarize the inter-spike membrane potential (ISI-Vm) to control levels to obtain AP recordings with matching ISI-Vm for comparison. The waveform analysis was carried on averaged APs with matching ISI-Vm. The threshold was detected using the time derivative of the membrane potential in the phase plots, as the point where the membrane derivative sharply rose or the values were higher than 10 V/s. When we compared the same set of APs, the mean of the threshold detected in individual APs (using spike 2 scripts) and the averaged APs was coincident, indicating that the averaging did not degrade the threshold detectability or measurement. The AP amplitude was taken as the difference in potential between the threshold and the AP peak. The AP half width was the duration of the spike at half amplitude. To estimate changes in the rate of the depolarizing and repolarizing AP phases the maximum and minimum values of the AP derivative were used respectively.

The basal AP firing rate was calculated for periods of at least 10 seconds of stable activity. To emulate synaptic evoked activity, hyperpolarizing or depolarizing current pulses (1 s duration) were injected. The maximum rebound frequency, was defined as the maximum instantaneous frequency attained over the first half second following the end of hyperpolarizing pulse injections of increasing intensity. To evaluate changes in the ability to fire repetitively at high frequency, the maximum repetitive firing frequency (MRF) was defined as the maximum steady-state frequency during the last 200 milliseconds of the spiking responses evoked by one second depolarizing current pulses of increasing intensity. Analysis was performed using programmable software: (Spike 2, CED), Igor (Wavemetrics Inc.) and further statistical analysis was carried out using Sigma Plot (SPSS Inc.). Data are presented as means ± SEM, sample size and type of statistical test used to assess significance are indicated in the main text and/or figure legends.
