# Author Correction: Kv1 potassium channels control action potential firing of putative GABAergic deep cerebellar nuclear neurons

**DOI:** 10.1038/s41598-020-68817-2

**Published:** 2020-07-14

**Authors:** Jessica Abigail Feria Pliego, Christine M. Pedroarena

**Affiliations:** 1 Graduate School of Cellular and Molecular Neurosciences, University of Tübingen, Tübingen, Germany; 2 Department for Cognitive Neurology, Hertie-Institute for Clinical Brain Research, 72076 Tübingen, Germany; 3 Systems Neurophysiology, Werner Reichardt Center for Integrative Neuroscience, University of Tübingen, 72076 Tübingen, Germany

Correction to:* Scientific Reports* 10.1038/s41598-020-63583-7, published online 24 April 2020

The original version of this Article contained errors.

Affiliation 3 was incorrectly given as ‘Systems Neurophysiology, Werner Reichardt Center for Integrative Neuroscience, University Tübingen, 72076, Tübingen, Germany’. The correct affiliation is listed below:

Systems Neurophysiology, Werner Reichardt Center for Integrative Neuroscience, University of Tübingen, 72076, Tübingen, Germany

In addition, the Acknowledgements section was incomplete.

“I thank Cornelius Schwarz for support for this research, alongside that from the Cognitive Neurology Department; Ute Grosshennig and Ursula Pascht for technical assistance; all members of the Cognitive Neurology Department for comments and helpful discussions.”

now reads:

“I thank Cornelius Schwarz for support for this research, alongside that from the Cognitive Neurology Department; Ute Grosshennig and Ursula Pascht for technical assistance; all members of the Cognitive Neurology Department for comments and helpful discussions. We acknowledge support by Open Access Publishing Fund of University of Tübingen.”

These errors have now been corrected in the HTML and PDF versions of the Article.

